# Eaton’s Domestic Practice for Parents and Nurses

**Published:** 1882-08

**Authors:** 


Eaton's Domestic Practice for Parents and Nurses. Illustrated. By Morton M. Eaton, M. D. pp. 686. Price, $3.50. New York and Philadelphia. Bœricke & Tafel. 1882.

Dr. Eaton's work is, it is said, written for parents and nurses. Our only hope is that they may never see it! Commencing with a dictionary and ending with a materia medica, embracing between these articles, on almost every "ill to which flesh is heir;" yet, in all this there is not one *true homœopathic prescription!* To our mind, these domestic works do much more harm than good; parents doctor their children only to waste valuable time and complicate the disease. But, if one must have such works, let them purchase Johnston's, or even better, Hering's (1851), taken from his seventh German edition.

